# Prevalence and association of MASLD in metabolically healthy young Asian Americans with obesity: A nationwide inpatient perspective (2019)

**DOI:** 10.1016/j.obpill.2025.100168

**Published:** 2025-02-18

**Authors:** Ahmad Alhomaid, Sukhjinder Chauhan, Yamini Katamreddy, Avideep Sidhu, Praveena Sunkara, Rupak Desai

**Affiliations:** a Nazareth Hospital, 2601 Holme Ave, Philadelphia, PA, 19152, USA; b Baptist Floyd Hospital, New Albany, USA; c Saint Michael's Medical Center, 111, Ave, Newark, NJ, 07102, USA; d Adesh Institute of Health & Medical Sciences, Bathinda, Punjab, 151001, India; e Passion Health Primary Care, 8195 Custer Rd Ste 110, Frisco, TX, 75035, USA; f Independent Researcher, Outcomes Research, Atlanta, GA, USA

**Keywords:** MASLD, MHO, Asian American, Young adults, NIS

## Abstract

**Background:**

Metabolic dysfunction-associated steatotic liver disease (MASLD) is a leading cause of chronic liver disease worldwide. Although the epidemiology of MASLD and its association with metabolically healthy obesity (MHO) is well-studied in the United States, data for Asian Americans with MHO is limited. We sought to evaluate the association of MASLD in young Asian American patients with MHO.

**Methods:**

This was a retrospective, matched cohort, database review of Asian American Individuals. After excluding adult hospitalizations with metabolic risk factors (hypertension, diabetes, or hyperlipidemia), we identified all National Inpatient Sample (2019) admissions with obesity (MHO) and MASLD using relevant ICD-10-CM codes. We matched (1:1) propensity scores for age, sex, household income, hospital location, and teaching status to obtain cohorts with and without obesity (MHO+) vs. (MHO-). Categorical and continuous data were compared using the Chi-square and Mann-Whitney U tests. The primary endpoint was the prevalence and adjusted multivariable odds/predictors of MASLD in (MHO+) vs. (MHO-) cohort.

**Results:**

In the adjusted multivariate regression for demographics, and comorbidities, the (MHO+) cohort was associated with higher odds of admissions with MASLD (OR 4.07, 95%CI 2.02–8.19, p ​< ​0.001). In addition, among the (MHO+) cohort, higher rates of MASLD-related hospitalizations were observed in males (OR 8.40, p ​< ​0.001), females (OR 2.69, p ​= ​0.025), high-income quartiles (OR 10.51, p ​< ​0.001), no prior bariatric surgery (OR 4.07, p ​< ​0.001), non-tobacco users(OR 4.16, p ​< ​0.001), and non-hypothyroid patients (OR 4.00, p ​< ​0.001) compared to the (MHO-) cohort. There was no statistically significant difference in the groups with low-income quartiles, tobacco use disorder, and hypothyroidism.

**Conclusion:**

This nationwide analysis demonstrates that (MHO+) is associated with a higher prevalence of MASLD. In the (MHO+) cohort, there was an association of MASLD with sex, high-income quartile, no prior bariatric surgery, non-tobacco use, and non-hypothyroidism. Further prospective multicenter studies are needed to evaluate the association of MASLD in (MHO+) patients with comorbid conditions.

## Graphical abstract

**Figure:**
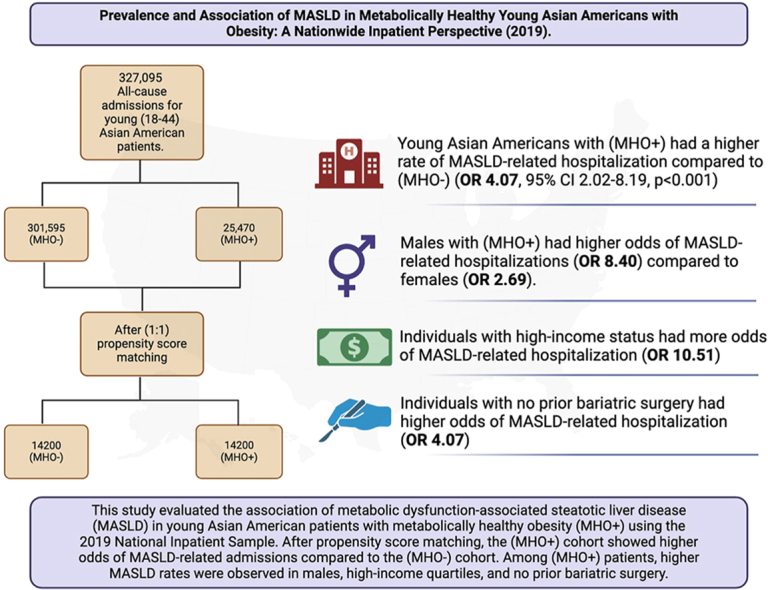

## 1 Introduction

Metabolic dysfunction-associated steatotic liver disease (MASLD) encompasses a spectrum of exceedingly common liver conditions ranging from simple steatosis to metabolic dysfunction-associated steatohepatitis non-alcoholic steatohepatitis (MASH) which may progress to cirrhosis and hepatocellular carcinoma (HCC) [1]. When compared to patients with obesity and concurrent metabolic syndrome features, individuals with metabolically healthy obesity (MHO+) typically exhibit the following pathophysiological characteristics: decreased accumulation of intra-abdominal visceral fat and ectopic fat for any given level of total adiposity, preserved insulin sensitivity, and lower degree of systemic and adipose tissue inflammation [2]. However, due to the complexity and variability of metabolic health, there is no clear cut definition for metabolic healthy obesity. Current proposed criteria include absence of metabolic syndrome (hyperglycemia, dyslipidemia and hypertension) [3]. Recently, observational studies have estimated the prevalence of MASLD in Asian American individuals to be around 40 % [4]. The same study also showed that Asian American have higher prevalence of MASLD in normal weight and individuals with morbid obesity even after using the lower BMI threshold defined by WHO [5]. Genetics play a major role in this since studies demonstrate that Asian individuals tend to have more visceral adipose tissue than other ethnic groups [6]. Other factors such as cultural influence and dietary choices could also have contributed. The importance of studying MASLD in young Asian populations is emphasized by research focusing on histological features in young Asian men. A study by Kim et al. [7]examined liver biopsies from 64 young MASLD patients and found that the vast majority (92.2 %) had MASH, including both borderline and definite cases. These findings highlight that MASLD in young Asian adults can present with complex and potentially more severe histological features than previously recognized. Given the high prevalence of MASH in this young population, there is a critical need for targeted research to understand the prevalence and association of MASLD in the young Asian populations in order to implement early detection and intervention strategies. Asian Americans are often underrepresented in research due to their frequent grouping with other ethnicities. Furthermore, the prevalence and risk of MASLD-related health issues among young Asian Americans with obesity but without obesity-related metabolic abnormalities (i.e., MHO) remains largely unexplored [8]. Given its severe health consequences and economical impact, studying the prevalence and impact of MASLD is crucial.

## 2 Methods

### 2.1 Data source

This was a retrospective, matched cohort, database review of Asian American Individuals.The study sample was acquired from the 2019’s National Inpatient Sample (NIS) database. The NIS database is the largest all-payer publicly available inpatient health care database in the United States. The NIS database includes more than 35 million weighted inpatient discharges and approximately 7 million unweighted inpatient discharges annually, which represents a 20 % sample from 1000 hospitals in at least 40 states across the United States [9].

### 2.2 Study population

Metabolic healthy obesity (MHO) was defined by excluding hospitalizations with a history of hypertension, diabetes mellitus, or hyperlipidemia. Specifically, we included all admissions among individuals of Asian descent with MASLD and then excluded encounters with concomitant hypertension, diabetes mellitus, or hyperlipidemia. The cohorts were subsequently categorized as those with obesity (MHO+) and those without obesity (MHO-). Codes for all cardiovascular comorbidities can be found in the supplemental tables provided in our study materials. The definition of Asian in our analysis encompasses all adults of Asian origin. The data source, however, does not provide a detailed breakdown of specific nationalities. For more information about the data elements, please refer to the HCUP documentation at this link-https://hcup-us.ahrq.gov/db/vars/race/nisnote.jsp.

After excluding adult hospitalizations with metabolic risk factors (hypertension, diabetes, or hyperlipidemia), 327,095 all-cause admissions for young Asian American patients (18–44 years old) were identified, including 301,595 [92.2 ​%] (MHO-) and 25,470 [7.8 ​%] (MHO+) patients. We used the International Classification of Diseases, 10th Revision Clinical Modification (ICD-10-CM) codes and the Revised Clinical Classifications Software (CCSR) to identify hospitalizations of individuals with MASLD, ICD-10-CM code K76.0. Since the NIS database does not contain patient identification data, we did not require approval from an institutional review board to carry out this investigation.

### 2.3 Study outcomes

We assessed and compared the baseline demographics, hospital-level characteristics and associated comorbidities in (MHO+) and (MHO-) related hospitalizations. The primary outcome of interest was the burden (frequency) of MASLD. Our secondary outcomes examined MASLD-related hospitalization, analyzing their association with gender, income status, history of bariatric surgery, use of tobacco and thyroid diseases.

### 2.4 Statistical analysis

We matched (1:1) propensity scores for age, sex, household income, hospital location, and teaching status to obtain cohorts with and without obesity (MHO+) vs. (MHO-). Categorical and continuous data were compared using the Chi-square and Mann-Whitney U tests. The primary endpoint was the prevalence and adjusted multivariable odds/predictors of MASLD in (MHO+) vs. (MHO-) cohort.

After excluding cardiovascular risk factors, we obtained 14200 (5.4 ​%) (MHO+). Propensity matching for age, sex, median household income, hospital location, and teaching status yielded 14200 patients in each cohort. Both cohorts had a comparable median age of 32 (IQR 27–36). The odds ratio (OR), 95 ​% confidence interval (CI), and P value were used to express the results of the logistic regression.

## 3 Results

### 3.1 Patient characteristics and outcomes

*Patient demographics:* After NIS weighing, we identified 327,095 all-cause admissions for young Asian American patients in the year 2019, of which 301595 [92.2 ​%] were without metabolically healthy obesity (MHO-) and 25470 [7.8 ​%] were with metabolically healthy obesity (MHO+). After excluding cardiovascular risk factors of hypertension and diabetes mellitus and propensity matching for age, sex, median household income, hospital location, and teaching status, we obtained 14200 [5.4 ​%] in each (MHO+) and (MHO-) cohort. Table 1 shows patient and hospital characteristics of hospital admission and outcomes. The median age was 32(IQR 27–36), and the majority of patients were females 24235 [85.3 %]. The majority of the patients were identified with median household income national quartile for patient zip code at 76-100th percentile [40 %]. Most patients were admitted to large urban teaching hospitals in the Western region.

**Table 1:** Patient characteristics and outcomes of the propensity score-matched cohorts of young Asian Americans with and without metabolically healthy obesity (Excluding patients with other metabolic risk factors such as hypertension, diabetes mellitus, and hyperlipidemia).

Patient Demographics		All Patients		(MHO-)		(MHO+)		*P* value
		N	%	N	%	N	%	
All patients		28400	100	14200	50	14200	50	–
Age(years) at admission, median (IQR)		32(27–36)	–	32(27–36)	–	32(27–36)	–	–
Sex	Male	4165	14.6	2160	15.2	2005	14.1	0.009
	Female	24235	85.3	12040	84.8	12195	85.9	
Median Household income nationwide quartile for zip code	0–25th percentile	3445	12.1	1705	12.0	1740	12.3	0.464
	26-50^th^ percentile	4990	17.5	2525	17.8	2465	17.4	
	51-75^th^ percentile	8545	30.0	4225	29.8	4320	30.4	
	76-100^th^ percentile	11420	40.2	5745	40.5	5675	40.0	
Bed size of the hospital	Small	5480	19.2	2480	17.5	3000	21.1	<0.001
	Medium	7895	27.7	3730	26.3	4165	29.3	
	Large	15025	52.9	7990	56.3	7035	49.5	
Location/Teaching status of the hospital	Rural	835	2.9	470	3.3	365	2.6	0.01
	Urban non-teaching	4235	14.9	2140	15.1	2095	14.8	
	Urban teaching	23330	82.1	11590	81.6	11740	82.7	
Region of the hospital	Northwest	3305	11.6	1640	11.5	1665	11.7	0.465
	Midwest	3565	12.5	1825	12.9	1740	12.3	
	South	5040	17.7	2500	17.6	2540	17.9	
	West	16580	58.3	8235	58.0	8255	58.1	
MASLD		280	0.9	60	0.4	220	1.5	<0.001

*Comorbidities:* Several comorbidities were identified in both (MHO+) and (MHO-) cohorts. Depression was seen in 425 [3 ​%] (MHO-) and 620 [4.4 ​%] (MHO+) patients. Cannabis use disorder was present in 340 [2.4 ​%] (MHO-) and 270 [1.9 ​%] (MHO+) patients. 125 [0.9 ​%] (MHO+) and 20 [0.1 ​%] (MHO-) patients had a history of bariatric surgery. 165 [1.2 ​%] (MHO+) and 360 [2.5 ​%] (MHO-) patients had cancer listed in their diagnosis. These groups also had a statistically significant association with tobacco use disorder, chronic pulmonary disease, hypothyroidism, and other thyroid disorders(p ​< ​0.05) which are shown in Table 2.

**Table 2:** Comorbidities in propensity score-matched cohorts of young Asian Americans with and without metabolically healthy obesity.

Comorbidities	All Patients		(MHO-)		(MHO+)		*P* value
	N	%	N	%	N	%	
All patients	28400	100	14200	50	14200	50	–
Human Immunodeficiency virus (HIV)	40	0.1	20	0.1	20	0.1	1.000
Acquired Immunodeficiency Syndrome (AIDS)	40	0.1	20	0.1	20	0.1	1.000
Depression	1045	3.6	425	3.0	620	4.4	<0.001
Peripheral vascular disease	105	0.36	50	0.4	55	0.4	0.625
Prior myocardial infarction	25	0.08	10	0.1	15	0.1	0.317
Drug abuse	825	2.9	410	2.9	415	2.9	0.860
Cannabis use disorder	610	2.1	340	2.4	270	1.9	0.004
Tobacco use disorder	2015	7.0	745	5.2	1270	8.9	<0.001
Chronic pulmonary disease	2000	7.0	595	4.2	1405	9.9	<0.001
Hypothyroidism	1595	5.6	605	4.3	990	7.0	<0.001
Other thyroid disorders	325	1.1	140	1.0	185	1.3	0.012
Prior TIA/stroke	65	0.2	25	0.2	40	0.3	0.063
Prior venous thromboembolism	185	0.6	80	0.6	105	0.7	0.065
History of bariatric surgery	145	0.5	20	0.1	125	0.9	<0.001
Cancer	525	1.8	360	2.5	165	1.2	<0.001

*Outcomes:* The prevalence of MASLD was noted in 220 [1.5 ​%] (MHO+) and 60 [0.4 ​%] (MHO-) patients shown in Table 1.

Odds of MASLD-related hospitalizations in young Asian American patients with (MHO+) and without MHO (MHO-):

In the adjusted multivariate regression for demographics and comorbidities, the (MHO+) cohort was associated with higher odds of admissions with MASLD (OR 4.07, 95 ​% CI 2.02–8.19, p ​< ​0.001). In addition, higher rates of MASLD-related hospitalizations were observed in males (OR 8.40, p ​< ​0.001) than females (OR 2.69, p ​= ​0.025), high-income quartiles (OR 10.51, p ​< ​0.001), no prior bariatric surgery (OR 4.07, p ​< ​0.001), non-tobacco users (OR 4.16, p ​< ​0.001), and non-hypothyroid patients (OR 4.00, p ​< ​0.001) compared to the (MHO-) cohort. There was no statistically significant difference in the groups with low-income quartiles, tobacco use disorder, and hypothyroidism shown in Table 3.

**Table 3:** Odds of hospitalizations with MASLD in young Asian American patients with (MHO+) and without (MHO-) metabolically healthy obesity.

	Odds Ratio (OR)	95 % Lower limit of CI	95 % Upper limit of CI	p value
Overall	4.07	2.02	8.19	<0.001
Male	8.40	2.45	28.77	0.001
Female	2.69	1.13	6.37	0.025
Low Income Quartile (0–25th percentile)	0.30	0.01	7.59	0.461
High Income Quartile (76-100^th^ percentile)	10.51	3.53	31.27	<0.001
No prior bariatric surgery	4.07	2.02	8.19	<0.001
No tobacco use disorder	4.16	1.89	9.14	<0.001
Tobacco use disorder	3.22	0.61	16.89	0.167
No hypothyroidism	4.00	2.00	7.98	<0.001
Hypothyroidism	1.65	0.14	20.02	0.692

## 4 Discussion

This nationwide analysis of young Asian American patients demonstrates a significant association between (MHO+) and MASLD. Our study demonstrates that young Asian Americans with (MHO+) had a higher rate of MASLD-related hospitalization compared to (MHO-), even after adjusting for demographics and comorbidities (OR 4.07, 95 ​% CI 2.02–8.19, p ​< ​0.001) The association between (MHO+) and MASLD in our study aligns with growing evidence that (MHO+) may not be as benign as the name suggests. These results are consistent with a large UK-based study cohort study conducted by Vusirikala et al. that showed (MHO+) patients were at significantly greater risk of developing MASLD compared to those with normal weight (Adjusted HR 6.92, 95 ​% CI 6.40–7.48) [10].The higher prevalence of MASLD in (MHO+) individuals may seem paradoxical given their lack of traditional metabolic risk factors. However, evidence demonstrates that even without metabolic abnormalities, (MHO+) individuals face significantly increased health risks compared to (MHO-). The literature supports this, showing that metabolic healthy obesity is associated with a 49 ​% higher risk of coronary heart disease, 7 ​% increased risk of cerebrovascular disease, and 96 ​% higher risk of heart failure [11]. Importantly, research suggests that the metabolically healthy status is often temporary rather than permanent. Long-term cohort studies have revealed that approximately 30–50 % of individuals initially classified as MHO eventually develop metabolic abnormalities over time [12].The higher MASLD admissions we observed might represent this transitional phase before other metabolic complications become clinically apparent. Our findings challenge the term “healthy obesity”. While (MHO+) individuals lack traditional metabolic risk factors, our results suggest that they still face increased health risks more than (MHO-) individuals. This is particularly concerning given the rising prevalence of obesity among Asian Americans and the known susceptibility of Asian populations to developing MASLD at lower BMI thresholds compared to other ethnicities [13]. This underscores the importance of addressing weight as a significant health risk, even in the absence of other metabolic disorders.

Interestingly, our analysis revealed significant gender differences in MASLD risk between (MHO+) and (MHO-) individuals. Males with (MHO+) had substantially higher odds of MASLD-related hospitalizations (OR 8.40) compared to females (OR 2.69). It resonates with existing literature on sex-specific metabolic variations and their roles in MASLD [14]. The underlying causes for these disparities could be attributed to hormonal differences affecting fat distribution and liver metabolism. Literature suggests that chronic liver disease including MASLD progresses more rapidly in men and postmenopausal women.^15 16^ In animal models, Estradiol, a potent antioxidant, suppresses hepatic fibrosis and inhibits hepatic stellate cell activation [15]. Shimizue et al. and Di Martino et al. suggested that Estradiol has a protective effect against liver fibrosis. This effect was particularly evident in Di Martino’s study, which showed decreased liver fibrosis in postmenopausal women who received hormone replacement therapy [16]. This gender disparity warrants further investigation and may have implications for targeted screening and prevention strategies.

It is believed that MASLD is associated with low-income status due to food scarcity and diet habits [17]. The association between MASLD and high-income quartiles (OR 10.51) in our study is a surprising finding. The results of our study may be attributed to individuals in high-income quartiles having more convenient access to healthcare and therefore the number of admissions is higher. Further exploration of socioeconomic status is needed to elucidate the underlying reasons for this relationship.

The connection between MASLD and no prior bariatric surgery (OR 4.07) is an interesting finding that encourages new directions.While the literature on this association is limited, these findings may open doors for research into how surgical interventions may interact with metabolic health.

The lack of association between MASLD and tobacco use or hypothyroidism in the (MHO+) group is noteworthy when compared with some previous studies in other populations [[18], [19], [20]], [[18], [19], [20]], [20,21].Regarding hypothyroidism, the regular use of thyroid hormone replacement therapy in diagnosed patients may help regulate metabolic processes, potentially reducing MASLD risk [22]. The lack of association with tobacco use could be attributed to multiple factors, including potential limitations in smoking status documentation, possible under-reporting or misclassification of smoking status in medical records, and differences in smoking patterns among Asian Americans with metabolically healthy obesity. This highlights the need for population-specific research to understand the unique risk factors and disease patterns in Asian Americans.

Our study highlights the complex nature of MHO, underscoring the fact that even in the absence of traditional metabolic risk factors, MASLD prevalence is markedly high. This complexity is in line with existing literature, suggesting that the current definition of MHO may be inadequate [23]. This definition is particularly important for populations like Asian Americans, who might be more prone to MASLD even without typical obesity-related metabolic abnormalities [24]. Our study aligns with literature and shows that Asian individuals are more susceptible to developing MASLD at a lower BMI than Caucasian individuals. Our results offer further proof for the need to reevaluate BMI cut-offs and other metabolic health criteria for different ethnicities. Additionally, this highlights the need for more comprehensive and ethnicity-specific health assessments in individuals with obesity, potentially including routine liver function tests and imaging studies to detect early signs of MASLD.

## 5 Limitations

While the large sample size and utilization of the National Inpatient Sample (NIS) database add strength to our study, several important limitations warrant mention. First, the cross-sectional nature of our study and lack of data on lifestyle factors, dietary patterns, physical activity, and genetic variables limit our ability to fully understand MASLD risk factors. Second, the NIS database does not capture outpatient medication use, including weight loss medications, which could explain some paradoxical findings between (MHO+) and (MHO-) groups. Third, the uneven gender distribution in our study population (85 ​% female vs 15 ​% male) may affect the interpretation of gender-specific MASLD risk differences. Finally, the database's standardized BMI cutoff of ≥30 for obesity may underestimate obesity prevalence in Asian Americans, who face increased metabolic risks at lower BMI thresholds (≥27.5 ​kg/m^2^). Our study aligns with calls in existing literature for a comprehensive, prospective approach to exploring MASLD in various ethnic and demographic groups [25].

## 6 Conclusion

Young Asian Americans with metabolic healthy obesity have significantly higher odds of MASLD-related hospitalizations compared to their counterparts without obesity. Our findings demonstrate a concerning association between obesity and MASLD in young Asian Americans and highlight the need for increased awareness of MASLD risk in this population, even in the absence of traditional metabolic risk factors. Our study also showed other significant discoveries. The existence of notable gender disparities, with (MHO+) males showing a substantially higher risk of MASLD-related hospitalization than females and the unexpected association of high-income status with increased MASLD risk in the (MHO+) cohort. These important findings warrant further investigation into gender-specific and socioeconomic factors. This NIS study highlights the importance of recognizing (MHO+) as a risk factor for MASLD among young Asian Americans. Future prospective studies are needed to further characterize the long-term liver-related outcomes in MHO individuals and to develop targeted prevention and screening strategies for this at-risk group.

## Key takeaways

• Young Asian Americans with (MHO+) are at higher risk of MASLD-related hospitalizations compared to individuals with (MHO-).
• Males have a higher rate of MASLD-related hospitalizations compared to females, underscoring the importance of hormonal differences.
• This study challenges the term “healthy obesity”, as individuals still face increased health risks even without metabolic dysfunction.

## Authors contributions

- Conception and design: RD, PS.

- Data collection and analysis: RD.

- Drafting and editing of Manuscript: RD, PS, AA, SC, YK, AS.

- Manuscript revision: RD, PS, AA.

## IRB approval

IRB approval is not **required** as this is a retrospective study and does not contain identifiable patient information.

## Declaration of artificial intelligence

During the preparation of this work the authors did not use AI.

## Source of funding

None.

## Conflict of interest

None.
