# Supplementary material for: Prevalence and association of MASLD in metabolically healthy young Asian Americans with obesity: A nationwide inpatient perspective (2019)
Source: Obes Pillars. 2025 Feb 18;13:100168. doi: 10.1016/j.obpill.2025.100168 (PMC11919439; doi:10.1016/j.obpill.2025.100168)
Supplement: Multimedia component 1 [file mmc1.docx]

**Supplementary Materials.1**

| **ICD-10 discharge codes for modifiable cardiovascular disease risk factors, including codes used under Elixhauser Software and Revised CCS** | |
| --- | --- |
| **Obesity** | |
| E6601 | Morbid (severe) obesity due to excess calories |
| E6609 | Other obesity due to excess calories |
| E661 | Drug-induced obesity |
| E662 | Morbid (severe) obesity with alveolar hypoventilation |
| E668 | Other obesity |
| E669 | Obesity, unspecified |
| O99210 | Obesity complicating pregnancy, unspecified trimester |
| O99211 | Obesity complicating pregnancy, first trimester |
| O99212 | Obesity complicating pregnancy, second trimester |
| O99213 | Obesity complicating pregnancy, third trimester |
| O99214 | Obesity complicating childbirth |
| O99215 | Obesity complicating the puerperium |
| R939 | Diagnostic imaging inconclusive due to excess body fat of patient |
| Z6830 | Body mass index [BMI] 30.0-30.9, adult |
| Z6831 | Body mass index [BMI] 31.0-31.9, adult |
| Z6832 | Body mass index [BMI] 32.0-32.9, adult |
| Z6833 | Body mass index [BMI] 33.0-33.9, adult |
| Z6834 | Body mass index [BMI] 34.0-34.9, adult |
| Z6835 | Body mass index [BMI] 35.0-35.9, adult |
| Z6836 | Body mass index [BMI] 36.0-36.9, adult |
| Z6837 | Body mass index [BMI] 37.0-37.9, adult |
| Z6838 | Body mass index [BMI] 38.0-38.9, adult |
| Z6839 | Body mass index [BMI] 39.0-39.9, adult |
| Z6841 | Body mass index [BMI] 40.0-44.9, adult |
| Z6842 | Body mass index [BMI] 45.0-49.9, adult |
| Z6843 | Body mass index [BMI] 50.0-59.9, adult |
| Z6844 | Body mass index [BMI] 60.0-69.9, adult |
| Z6845 | Body mass index [BMI] 70 or greater, adult |

| **Uncomplicated Hypertension** | |
| --- | --- |
| I10 | Essential (primary) hypertension |
| I160 | Hypertensive urgency |
| I169 | Hypertensive crisis, unspecified |
| I1A0 | Resistant hypertension |
| O10011 | Pre-existing essential hypertension complicating pregnancy, first trimester |
| O10012 | Pre-existing essential hypertension complicating pregnancy, second trimester |
| O10013 | Pre-existing essential hypertension complicating pregnancy, third trimester |
| O10019 | Pre-existing essential hypertension complicating pregnancy, unspecified trimester |
| O1002 | Pre-existing essential hypertension complicating childbirth |
| O1003 | Pre-existing essential hypertension complicating the puerperium |

| **Complicated Hypertension** | |
| --- | --- |
| H35031 | Hypertensive retinopathy, right eye |
| H35032 | Hypertensive retinopathy, left eye |
| H35033 | Hypertensive retinopathy, bilateral |
| H35039 | Hypertensive retinopathy, unspecified eye |
| I110 | Hypertensive heart disease with heart failure |
| I119 | Hypertensive heart disease without heart failure |
| I120 | Hypertensive chronic kidney disease with stage 5 chronic kidney disease or end stage renal disease |
| I129 | Hypertensive chronic kidney disease with stage 1 through stage 4 chronic kidney disease, or unspecified chronic kidney disease |
| I130 | Hypertensive heart and chronic kidney disease with heart failure and stage 1 through stage 4 chronic kidney disease, or unspecified chronic kidney disease |
| I1310 | Hypertensive heart and chronic kidney disease without heart failure, with stage 1 through stage 4 chronic kidney disease, or unspecified chronic kidney disease |
| I1311 | Hypertensive heart and chronic kidney disease without heart failure, with stage 5 chronic kidney disease, or end stage renal disease |
| I132 | Hypertensive heart and chronic kidney disease with heart failure and with stage 5 chronic kidney disease, or end stage renal disease |
| I150 | Renovascular hypertension |
| I151 | Hypertension secondary to other renal disorders |
| I152 | Hypertension secondary to endocrine disorders |
| I158 | Other secondary hypertension |
| I159 | Secondary hypertension, unspecified |
| I161 | Hypertensive emergency |
| I674 | Hypertensive encephalopathy |
| O10111 | Pre-existing hypertensive heart disease complicating pregnancy, first trimester |
| O10112 | Pre-existing hypertensive heart disease complicating pregnancy, second trimester |
| O10113 | Pre-existing hypertensive heart disease complicating pregnancy, third trimester |
| O10119 | Pre-existing hypertensive heart disease complicating pregnancy, unspecified trimester |
| O1012 | Pre-existing hypertensive heart disease complicating childbirth |
| O1013 | Pre-existing hypertensive heart disease complicating the puerperium |
| O10211 | Pre-existing hypertensive chronic kidney disease complicating pregnancy, first trimester |
| O10212 | Pre-existing hypertensive chronic kidney disease complicating pregnancy, second trimester |
| O10213 | Pre-existing hypertensive chronic kidney disease complicating pregnancy, third trimester |
| O10219 | Pre-existing hypertensive chronic kidney disease complicating pregnancy, unspecified trimester |
| O1022 | Pre-existing hypertensive chronic kidney disease complicating childbirth |
| O1023 | Pre-existing hypertensive chronic kidney disease complicating the puerperium |
| O10311 | Pre-existing hypertensive heart and chronic kidney disease complicating pregnancy, first trimester |
| O10312 | Pre-existing hypertensive heart and chronic kidney disease complicating pregnancy, second trimester |
| O10313 | Pre-existing hypertensive heart and chronic kidney disease complicating pregnancy, third trimester |
| O10319 | Pre-existing hypertensive heart and chronic kidney disease complicating pregnancy, unspecified trimester |
| O1032 | Pre-existing hypertensive heart and chronic kidney disease complicating childbirth |
| O1033 | Pre-existing hypertensive heart and chronic kidney disease complicating the puerperium |
| O10411 | Pre-existing secondary hypertension complicating pregnancy, first trimester |
| O10412 | Pre-existing secondary hypertension complicating pregnancy, second trimester |
| O10413 | Pre-existing secondary hypertension complicating pregnancy, third trimester |
| O10419 | Pre-existing secondary hypertension complicating pregnancy, unspecified trimester |
| O1042 | Pre-existing secondary hypertension complicating childbirth |
| O1043 | Pre-existing secondary hypertension complicating the puerperium |
| O10911 | Unspecified pre-existing hypertension complicating pregnancy, first trimester |
| O10912 | Unspecified pre-existing hypertension complicating pregnancy, second trimester |
| O10913 | Unspecified pre-existing hypertension complicating pregnancy, third trimester |
| O10919 | Unspecified pre-existing hypertension complicating pregnancy, unspecified trimester |
| O1092 | Unspecified pre-existing hypertension complicating childbirth |
| O1093 | Unspecified pre-existing hypertension complicating the puerperium |
| O111 | Pre-existing hypertension with pre-eclampsia, first trimester |
| O112 | Pre-existing hypertension with pre-eclampsia, second trimester |
| O113 | Pre-existing hypertension with pre-eclampsia, third trimester |
| O114 | Pre-existing hypertension with pre-eclampsia, complicating childbirth |
| O115 | Pre-existing hypertension with pre-eclampsia, complicating the puerperium |
| O119 | Pre-existing hypertension with pre-eclampsia, unspecified trimester |
| O161 | Unspecified maternal hypertension, first trimester |
| O162 | Unspecified maternal hypertension, second trimester |
| O163 | Unspecified maternal hypertension, third trimester |
| O164 | Unspecified maternal hypertension, complicating childbirth |
| O165 | Unspecified maternal hypertension, complicating the puerperium |
| O169 | Unspecified maternal hypertension, unspecified trimester |

| **Uncomplicated Diabetes Mellitus** | |
| --- | --- |
| E0800 | Diabetes mellitus due to underlying condition with hyperosmolarity without nonketotic hyperglycemic-hyperosmolar coma (NKHHC) |
| E0801 | Diabetes mellitus due to underlying condition with hyperosmolarity with coma |
| E0810 | Diabetes mellitus due to underlying condition with ketoacidosis without coma |
| E0811 | Diabetes mellitus due to underlying condition with ketoacidosis with coma |
| E089 | Diabetes mellitus due to underlying condition without complications |
| E0900 | Drug or chemical induced diabetes mellitus with hyperosmolarity without nonketotic hyperglycemic-hyperosmolar coma (NKHHC) |
| E0901 | Drug or chemical induced diabetes mellitus with hyperosmolarity with coma |
| E0910 | Drug or chemical induced diabetes mellitus with ketoacidosis without coma |
| E0911 | Drug or chemical induced diabetes mellitus with ketoacidosis with coma |
| E099 | Drug or chemical induced diabetes mellitus without complications |
| E1010 | Type 1 diabetes mellitus with ketoacidosis without coma |
| E1011 | Type 1 diabetes mellitus with ketoacidosis with coma |
| E109 | Type 1 diabetes mellitus without complications |
| E1100 | Type 2 diabetes mellitus with hyperosmolarity without nonketotic hyperglycemic-hyperosmolar coma (NKHHC) |
| E1101 | Type 2 diabetes mellitus with hyperosmolarity with coma |
| E1110 | Type 2 diabetes mellitus with ketoacidosis without coma |
| E1111 | Type 2 diabetes mellitus with ketoacidosis with coma |
| E119 | Type 2 diabetes mellitus without complications |
| E1300 | Other specified diabetes mellitus with hyperosmolarity without nonketotic hyperglycemic-hyperosmolar coma (NKHHC) |
| E1301 | Other specified diabetes mellitus with hyperosmolarity with coma |
| E1310 | Other specified diabetes mellitus with ketoacidosis without coma |
| E1311 | Other specified diabetes mellitus with ketoacidosis with coma |
| E139 | Other specified diabetes mellitus without complications |
| O24011 | Pre-existing type 1 diabetes mellitus, in pregnancy, first trimester |
| O24012 | Pre-existing type 1 diabetes mellitus, in pregnancy, second trimester |
| O24013 | Pre-existing type 1 diabetes mellitus, in pregnancy, third trimester |
| O24019 | Pre-existing type 1 diabetes mellitus, in pregnancy, unspecified trimester |
| O2402 | Pre-existing type 1 diabetes mellitus, in childbirth |
| O2403 | Pre-existing type 1 diabetes mellitus, in the puerperium |
| O24111 | Pre-existing type 2 diabetes mellitus, in pregnancy, first trimester |
| O24112 | Pre-existing type 2 diabetes mellitus, in pregnancy, second trimester |
| O24113 | Pre-existing type 2 diabetes mellitus, in pregnancy, third trimester |
| O24119 | Pre-existing type 2 diabetes mellitus, in pregnancy, unspecified trimester |
| O2412 | Pre-existing type 2 diabetes mellitus, in childbirth |
| O2413 | Pre-existing type 2 diabetes mellitus, in the puerperium |
| O24311 | Unspecified pre-existing diabetes mellitus in pregnancy, first trimester |
| O24312 | Unspecified pre-existing diabetes mellitus in pregnancy, second trimester |
| O24313 | Unspecified pre-existing diabetes mellitus in pregnancy, third trimester |
| O24319 | Unspecified pre-existing diabetes mellitus in pregnancy, unspecified trimester |
| O2432 | Unspecified pre-existing diabetes mellitus in childbirth |
| O2433 | Unspecified pre-existing diabetes mellitus in the puerperium |
| O24410 | Gestational diabetes mellitus in pregnancy, diet controlled |
| O24414 | Gestational diabetes mellitus in pregnancy, insulin controlled |
| O24415 | Gestational diabetes mellitus in pregnancy, controlled by oral hypoglycemic drugs |
| O24419 | Gestational diabetes mellitus in pregnancy, unspecified control |
| O24420 | Gestational diabetes mellitus in childbirth, diet controlled |
| O24424 | Gestational diabetes mellitus in childbirth, insulin controlled |
| O24425 | Gestational diabetes mellitus in childbirth, controlled by oral hypoglycemic drugs |
| O24429 | Gestational diabetes mellitus in childbirth, unspecified control |
| O24430 | Gestational diabetes mellitus in the puerperium, diet controlled |
| O24434 | Gestational diabetes mellitus in the puerperium, insulin controlled |
| O24435 | Gestational diabetes mellitus in puerperium, controlled by oral hypoglycemic drugs |
| O24439 | Gestational diabetes mellitus in the puerperium, unspecified control |
| O24811 | Other pre-existing diabetes mellitus in pregnancy, first trimester |
| O24812 | Other pre-existing diabetes mellitus in pregnancy, second trimester |
| O24813 | Other pre-existing diabetes mellitus in pregnancy, third trimester |
| O24819 | Other pre-existing diabetes mellitus in pregnancy, unspecified trimester |
| O2482 | Other pre-existing diabetes mellitus in childbirth |
| O2483 | Other pre-existing diabetes mellitus in the puerperium |
| O24911 | Unspecified diabetes mellitus in pregnancy, first trimester |
| O24912 | Unspecified diabetes mellitus in pregnancy, second trimester |
| O24913 | Unspecified diabetes mellitus in pregnancy, third trimester |
| O24919 | Unspecified diabetes mellitus in pregnancy, unspecified trimester |
| O2492 | Unspecified diabetes mellitus in childbirth |
| O2493 | Unspecified diabetes mellitus in the puerperium |

| **Complicated Diabetes Mellitus** | |
| --- | --- |
| E0821 | Diabetes mellitus due to underlying condition with diabetic nephropathy |
| E0822 | Diabetes mellitus due to underlying condition with diabetic chronic kidney disease |
| E0829 | Diabetes mellitus due to underlying condition with other diabetic kidney complication |
| E08311 | Diabetes mellitus due to underlying condition with unspecified diabetic retinopathy with macular edema |
| E08319 | Diabetes mellitus due to underlying condition with unspecified diabetic retinopathy without macular edema |
| E08321 | Diabetes mellitus due to underlying condition with mild nonproliferative diabetic retinopathy with macular edema |
| E083211 | Diabetes mellitus due to underlying condition with mild nonproliferative diabetic retinopathy with macular edema, right eye |
| E083212 | Diabetes mellitus due to underlying condition with mild nonproliferative diabetic retinopathy with macular edema, left eye |
| E083213 | Diabetes mellitus due to underlying condition with mild nonproliferative diabetic retinopathy with macular edema, bilateral |
| E083219 | Diabetes mellitus due to underlying condition with mild nonproliferative diabetic retinopathy with macular edema, unspecified eye |
| E08329 | Diabetes mellitus due to underlying condition with mild nonproliferative diabetic retinopathy without macular edema |
| E083291 | Diabetes mellitus due to underlying condition with mild nonproliferative diabetic retinopathy without macular edema, right eye |
| E083292 | Diabetes mellitus due to underlying condition with mild nonproliferative diabetic retinopathy without macular edema, left eye |
| E083293 | Diabetes mellitus due to underlying condition with mild nonproliferative diabetic retinopathy without macular edema, bilateral |
| E083299 | Diabetes mellitus due to underlying condition with mild nonproliferative diabetic retinopathy without macular edema, unspecified eye |
| E08331 | Diabetes mellitus due to underlying condition with moderate nonproliferative diabetic retinopathy with macular edema |
| E083311 | Diabetes mellitus due to underlying condition with moderate nonproliferative diabetic retinopathy with macular edema, right eye |
| E083312 | Diabetes mellitus due to underlying condition with moderate nonproliferative diabetic retinopathy with macular edema, left eye |
| E083313 | Diabetes mellitus due to underlying condition with moderate nonproliferative diabetic retinopathy with macular edema, bilateral |
| E083319 | Diabetes mellitus due to underlying condition with moderate nonproliferative diabetic retinopathy with macular edema, unspecified eye |
| E08339 | Diabetes mellitus due to underlying condition with moderate nonproliferative diabetic retinopathy without macular edema |
| E083391 | Diabetes mellitus due to underlying condition with moderate nonproliferative diabetic retinopathy without macular edema, right eye |
| E083392 | Diabetes mellitus due to underlying condition with moderate nonproliferative diabetic retinopathy without macular edema, left eye |
| E083393 | Diabetes mellitus due to underlying condition with moderate nonproliferative diabetic retinopathy without macular edema, bilateral |
| E083399 | Diabetes mellitus due to underlying condition with moderate nonproliferative diabetic retinopathy without macular edema, unspecified eye |
| E08341 | Diabetes mellitus due to underlying condition with severe nonproliferative diabetic retinopathy with macular edema |
| E083411 | Diabetes mellitus due to underlying condition with severe nonproliferative diabetic retinopathy with macular edema, right eye |
| E083412 | Diabetes mellitus due to underlying condition with severe nonproliferative diabetic retinopathy with macular edema, left eye |
| E083413 | Diabetes mellitus due to underlying condition with severe nonproliferative diabetic retinopathy with macular edema, bilateral |
| E083419 | Diabetes mellitus due to underlying condition with severe nonproliferative diabetic retinopathy with macular edema, unspecified eye |
| E08349 | Diabetes mellitus due to underlying condition with severe nonproliferative diabetic retinopathy without macular edema |
| E083491 | Diabetes mellitus due to underlying condition with severe nonproliferative diabetic retinopathy without macular edema, right eye |
| E083492 | Diabetes mellitus due to underlying condition with severe nonproliferative diabetic retinopathy without macular edema, left eye |
| E083493 | Diabetes mellitus due to underlying condition with severe nonproliferative diabetic retinopathy without macular edema, bilateral |
| E083499 | Diabetes mellitus due to underlying condition with severe nonproliferative diabetic retinopathy without macular edema, unspecified eye |
| E08351 | Diabetes mellitus due to underlying condition with proliferative diabetic retinopathy with macular edema |
| E083511 | Diabetes mellitus due to underlying condition with proliferative diabetic retinopathy with macular edema, right eye |
| E083512 | Diabetes mellitus due to underlying condition with proliferative diabetic retinopathy with macular edema, left eye |
| E083513 | Diabetes mellitus due to underlying condition with proliferative diabetic retinopathy with macular edema, bilateral |
| E083519 | Diabetes mellitus due to underlying condition with proliferative diabetic retinopathy with macular edema, unspecified eye |
| E083521 | Diabetes mellitus due to underlying condition with proliferative diabetic retinopathy with traction retinal detachment involving the macula, right eye |
| E083522 | Diabetes mellitus due to underlying condition with proliferative diabetic retinopathy with traction retinal detachment involving the macula, left eye |
| E083523 | Diabetes mellitus due to underlying condition with proliferative diabetic retinopathy with traction retinal detachment involving the macula, bilateral |
| E083529 | Diabetes mellitus due to underlying condition with proliferative diabetic retinopathy with traction retinal detachment involving the macula, unspecified eye |
| E083531 | Diabetes mellitus due to underlying condition with proliferative diabetic retinopathy with traction retinal detachment not involving the macula, right eye |
| E083532 | Diabetes mellitus due to underlying condition with proliferative diabetic retinopathy with traction retinal detachment not involving the macula, left eye |
| E083533 | Diabetes mellitus due to underlying condition with proliferative diabetic retinopathy with traction retinal detachment not involving the macula, bilateral |
| E083539 | Diabetes mellitus due to underlying condition with proliferative diabetic retinopathy with traction retinal detachment not involving the macula, unspecified eye |
| E083541 | Diabetes mellitus due to underlying condition with proliferative diabetic retinopathy with combined traction retinal detachment and rhegmatogenous retinal detachment, right eye |
| E083542 | Diabetes mellitus due to underlying condition with proliferative diabetic retinopathy with combined traction retinal detachment and rhegmatogenous retinal detachment, left eye |
| E083543 | Diabetes mellitus due to underlying condition with proliferative diabetic retinopathy with combined traction retinal detachment and rhegmatogenous retinal detachment, bilateral |
| E083549 | Diabetes mellitus due to underlying condition with proliferative diabetic retinopathy with combined traction retinal detachment and rhegmatogenous retinal detachment, unspecified eye |
| E083551 | Diabetes mellitus due to underlying condition with stable proliferative diabetic retinopathy, right eye |
| E083552 | Diabetes mellitus due to underlying condition with stable proliferative diabetic retinopathy, left eye |
| E083553 | Diabetes mellitus due to underlying condition with stable proliferative diabetic retinopathy, bilateral |
| E083559 | Diabetes mellitus due to underlying condition with stable proliferative diabetic retinopathy, unspecified eye |
| E08359 | Diabetes mellitus due to underlying condition with proliferative diabetic retinopathy without macular edema |
| E083591 | Diabetes mellitus due to underlying condition with proliferative diabetic retinopathy without macular edema, right eye |
| E083592 | Diabetes mellitus due to underlying condition with proliferative diabetic retinopathy without macular edema, left eye |
| E083593 | Diabetes mellitus due to underlying condition with proliferative diabetic retinopathy without macular edema, bilateral |
| E083599 | Diabetes mellitus due to underlying condition with proliferative diabetic retinopathy without macular edema, unspecified eye |
| E0836 | Diabetes mellitus due to underlying condition with diabetic cataract |
| E0837X1 | Diabetes mellitus due to underlying condition with diabetic macular edema, resolved following treatment, right eye |
| E0837X2 | Diabetes mellitus due to underlying condition with diabetic macular edema, resolved following treatment, left eye |
| E0837X3 | Diabetes mellitus due to underlying condition with diabetic macular edema, resolved following treatment, bilateral |
| E0837X9 | Diabetes mellitus due to underlying condition with diabetic macular edema, resolved following treatment, unspecified eye |
| E0839 | Diabetes mellitus due to underlying condition with other diabetic ophthalmic complication |
| E0840 | Diabetes mellitus due to underlying condition with diabetic neuropathy, unspecified |
| E0841 | Diabetes mellitus due to underlying condition with diabetic mononeuropathy |
| E0842 | Diabetes mellitus due to underlying condition with diabetic polyneuropathy |
| E0843 | Diabetes mellitus due to underlying condition with diabetic autonomic (poly)neuropathy |
| E0844 | Diabetes mellitus due to underlying condition with diabetic amyotrophy |
| E0849 | Diabetes mellitus due to underlying condition with other diabetic neurological complication |
| E0851 | Diabetes mellitus due to underlying condition with diabetic peripheral angiopathy without gangrene |
| E0852 | Diabetes mellitus due to underlying condition with diabetic peripheral angiopathy with gangrene |
| E0859 | Diabetes mellitus due to underlying condition with other circulatory complications |
| E08610 | Diabetes mellitus due to underlying condition with diabetic neuropathic arthropathy |
| E08618 | Diabetes mellitus due to underlying condition with other diabetic arthropathy |
| E08620 | Diabetes mellitus due to underlying condition with diabetic dermatitis |
| E08621 | Diabetes mellitus due to underlying condition with foot ulcer |
| E08622 | Diabetes mellitus due to underlying condition with other skin ulcer |
| E08628 | Diabetes mellitus due to underlying condition with other skin complications |
| E08630 | Diabetes mellitus due to underlying condition with periodontal disease |
| E08638 | Diabetes mellitus due to underlying condition with other oral complications |
| E08641 | Diabetes mellitus due to underlying condition with hypoglycemia with coma |
| E08649 | Diabetes mellitus due to underlying condition with hypoglycemia without coma |
| E0865 | Diabetes mellitus due to underlying condition with hyperglycemia |
| E0869 | Diabetes mellitus due to underlying condition with other specified complication |
| E088 | Diabetes mellitus due to underlying condition with unspecified complications |
| E0921 | Drug or chemical induced diabetes mellitus with diabetic nephropathy |
| E0922 | Drug or chemical induced diabetes mellitus with diabetic chronic kidney disease |
| E0929 | Drug or chemical induced diabetes mellitus with other diabetic kidney complication |
| E09311 | Drug or chemical induced diabetes mellitus with unspecified diabetic retinopathy with macular edema |
| E09319 | Drug or chemical induced diabetes mellitus with unspecified diabetic retinopathy without macular edema |
| E09321 | Drug or chemical induced diabetes mellitus with mild nonproliferative diabetic retinopathy with macular edema |
| E093211 | Drug or chemical induced diabetes mellitus with mild nonproliferative diabetic retinopathy with macular edema, right eye |
| E093212 | Drug or chemical induced diabetes mellitus with mild nonproliferative diabetic retinopathy with macular edema, left eye |
| E093213 | Drug or chemical induced diabetes mellitus with mild nonproliferative diabetic retinopathy with macular edema, bilateral |
| E093219 | Drug or chemical induced diabetes mellitus with mild nonproliferative diabetic retinopathy with macular edema, unspecified eye |
| E09329 | Drug or chemical induced diabetes mellitus with mild nonproliferative diabetic retinopathy without macular edema |
| E093291 | Drug or chemical induced diabetes mellitus with mild nonproliferative diabetic retinopathy without macular edema, right eye |
| E093292 | Drug or chemical induced diabetes mellitus with mild nonproliferative diabetic retinopathy without macular edema, left eye |
| E093293 | Drug or chemical induced diabetes mellitus with mild nonproliferative diabetic retinopathy without macular edema, bilateral |
| E093299 | Drug or chemical induced diabetes mellitus with mild nonproliferative diabetic retinopathy without macular edema, unspecified eye |
| E09331 | Drug or chemical induced diabetes mellitus with moderate nonproliferative diabetic retinopathy with macular edema |
| E093311 | Drug or chemical induced diabetes mellitus with moderate nonproliferative diabetic retinopathy with macular edema, right eye |
| E093312 | Drug or chemical induced diabetes mellitus with moderate nonproliferative diabetic retinopathy with macular edema, left eye |
| E093313 | Drug or chemical induced diabetes mellitus with moderate nonproliferative diabetic retinopathy with macular edema, bilateral |
| E093319 | Drug or chemical induced diabetes mellitus with moderate nonproliferative diabetic retinopathy with macular edema, unspecified eye |
| E09339 | Drug or chemical induced diabetes mellitus with moderate nonproliferative diabetic retinopathy without macular edema |
| E093391 | Drug or chemical induced diabetes mellitus with moderate nonproliferative diabetic retinopathy without macular edema, right eye |
| E093392 | Drug or chemical induced diabetes mellitus with moderate nonproliferative diabetic retinopathy without macular edema, left eye |
| E093393 | Drug or chemical induced diabetes mellitus with moderate nonproliferative diabetic retinopathy without macular edema, bilateral |
| E093399 | Drug or chemical induced diabetes mellitus with moderate nonproliferative diabetic retinopathy without macular edema, unspecified eye |
| E09341 | Drug or chemical induced diabetes mellitus with severe nonproliferative diabetic retinopathy with macular edema |
| E093411 | Drug or chemical induced diabetes mellitus with severe nonproliferative diabetic retinopathy with macular edema, right eye |
| E093412 | Drug or chemical induced diabetes mellitus with severe nonproliferative diabetic retinopathy with macular edema, left eye |
| E093413 | Drug or chemical induced diabetes mellitus with severe nonproliferative diabetic retinopathy with macular edema, bilateral |
| E093419 | Drug or chemical induced diabetes mellitus with severe nonproliferative diabetic retinopathy with macular edema, unspecified eye |
| E09349 | Drug or chemical induced diabetes mellitus with severe nonproliferative diabetic retinopathy without macular edema |
| E093491 | Drug or chemical induced diabetes mellitus with severe nonproliferative diabetic retinopathy without macular edema, right eye |
| E093492 | Drug or chemical induced diabetes mellitus with severe nonproliferative diabetic retinopathy without macular edema, left eye |
| E093493 | Drug or chemical induced diabetes mellitus with severe nonproliferative diabetic retinopathy without macular edema, bilateral |
| E093499 | Drug or chemical induced diabetes mellitus with severe nonproliferative diabetic retinopathy without macular edema, unspecified eye |
| E09351 | Drug or chemical induced diabetes mellitus with proliferative diabetic retinopathy with macular edema |
| E093511 | Drug or chemical induced diabetes mellitus with proliferative diabetic retinopathy with macular edema, right eye |
| E093512 | Drug or chemical induced diabetes mellitus with proliferative diabetic retinopathy with macular edema, left eye |
| E093513 | Drug or chemical induced diabetes mellitus with proliferative diabetic retinopathy with macular edema, bilateral |
| E093519 | Drug or chemical induced diabetes mellitus with proliferative diabetic retinopathy with macular edema, unspecified eye |
| E093521 | Drug or chemical induced diabetes mellitus with proliferative diabetic retinopathy with traction retinal detachment involving the macula, right eye |
| E093522 | Drug or chemical induced diabetes mellitus with proliferative diabetic retinopathy with traction retinal detachment involving the macula, left eye |
| E093523 | Drug or chemical induced diabetes mellitus with proliferative diabetic retinopathy with traction retinal detachment involving the macula, bilateral |
| E093529 | Drug or chemical induced diabetes mellitus with proliferative diabetic retinopathy with traction retinal detachment involving the macula, unspecified eye |
| E093531 | Drug or chemical induced diabetes mellitus with proliferative diabetic retinopathy with traction retinal detachment not involving the macula, right eye |
| E093532 | Drug or chemical induced diabetes mellitus with proliferative diabetic retinopathy with traction retinal detachment not involving the macula, left eye |
| E093533 | Drug or chemical induced diabetes mellitus with proliferative diabetic retinopathy with traction retinal detachment not involving the macula, bilateral |
| E093539 | Drug or chemical induced diabetes mellitus with proliferative diabetic retinopathy with traction retinal detachment not involving the macula, unspecified eye |
| E093541 | Drug or chemical induced diabetes mellitus with proliferative diabetic retinopathy with combined traction retinal detachment and rhegmatogenous retinal detachment, right eye |
| E093542 | Drug or chemical induced diabetes mellitus with proliferative diabetic retinopathy with combined traction retinal detachment and rhegmatogenous retinal detachment, left eye |
| E093543 | Drug or chemical induced diabetes mellitus with proliferative diabetic retinopathy with combined traction retinal detachment and rhegmatogenous retinal detachment, bilateral |
| E093549 | Drug or chemical induced diabetes mellitus with proliferative diabetic retinopathy with combined traction retinal detachment and rhegmatogenous retinal detachment, unspecified eye |
| E093551 | Drug or chemical induced diabetes mellitus with stable proliferative diabetic retinopathy, right eye |
| E093552 | Drug or chemical induced diabetes mellitus with stable proliferative diabetic retinopathy, left eye |
| E093553 | Drug or chemical induced diabetes mellitus with stable proliferative diabetic retinopathy, bilateral |
| E093559 | Drug or chemical induced diabetes mellitus with stable proliferative diabetic retinopathy, unspecified eye |
| E09359 | Drug or chemical induced diabetes mellitus with proliferative diabetic retinopathy without macular edema |
| E093591 | Drug or chemical induced diabetes mellitus with proliferative diabetic retinopathy without macular edema, right eye |
| E093592 | Drug or chemical induced diabetes mellitus with proliferative diabetic retinopathy without macular edema, left eye |
| E093593 | Drug or chemical induced diabetes mellitus with proliferative diabetic retinopathy without macular edema, bilateral |
| E093599 | Drug or chemical induced diabetes mellitus with proliferative diabetic retinopathy without macular edema, unspecified eye |
| E0936 | Drug or chemical induced diabetes mellitus with diabetic cataract |
| E0937X1 | Drug or chemical induced diabetes mellitus with diabetic macular edema, resolved following treatment, right eye |
| E0937X2 | Drug or chemical induced diabetes mellitus with diabetic macular edema, resolved following treatment, left eye |
| E0937X3 | Drug or chemical induced diabetes mellitus with diabetic macular edema, resolved following treatment, bilateral |
| E0937X9 | Drug or chemical induced diabetes mellitus with diabetic macular edema, resolved following treatment, unspecified eye |
| E0939 | Drug or chemical induced diabetes mellitus with other diabetic ophthalmic complication |
| E0940 | Drug or chemical induced diabetes mellitus with neurological complications with diabetic neuropathy, unspecified |
| E0941 | Drug or chemical induced diabetes mellitus with neurological complications with diabetic mononeuropathy |
| E0942 | Drug or chemical induced diabetes mellitus with neurological complications with diabetic polyneuropathy |
| E0943 | Drug or chemical induced diabetes mellitus with neurological complications with diabetic autonomic (poly)neuropathy |
| E0944 | Drug or chemical induced diabetes mellitus with neurological complications with diabetic amyotrophy |
| E0949 | Drug or chemical induced diabetes mellitus with neurological complications with other diabetic neurological complication |
| E0951 | Drug or chemical induced diabetes mellitus with diabetic peripheral angiopathy without gangrene |
| E0952 | Drug or chemical induced diabetes mellitus with diabetic peripheral angiopathy with gangrene |
| E0959 | Drug or chemical induced diabetes mellitus with other circulatory complications |
| E09610 | Drug or chemical induced diabetes mellitus with diabetic neuropathic arthropathy |
| E09618 | Drug or chemical induced diabetes mellitus with other diabetic arthropathy |
| E09620 | Drug or chemical induced diabetes mellitus with diabetic dermatitis |
| E09621 | Drug or chemical induced diabetes mellitus with foot ulcer |
| E09622 | Drug or chemical induced diabetes mellitus with other skin ulcer |
| E09628 | Drug or chemical induced diabetes mellitus with other skin complications |
| E09630 | Drug or chemical induced diabetes mellitus with periodontal disease |
| E09638 | Drug or chemical induced diabetes mellitus with other oral complications |
| E09641 | Drug or chemical induced diabetes mellitus with hypoglycemia with coma |
| E09649 | Drug or chemical induced diabetes mellitus with hypoglycemia without coma |
| E0965 | Drug or chemical induced diabetes mellitus with hyperglycemia |
| E0969 | Drug or chemical induced diabetes mellitus with other specified complication |
| E098 | Drug or chemical induced diabetes mellitus with unspecified complications |
| E1021 | Type 1 diabetes mellitus with diabetic nephropathy |
| E1022 | Type 1 diabetes mellitus with diabetic chronic kidney disease |
| E1029 | Type 1 diabetes mellitus with other diabetic kidney complication |
| E10311 | Type 1 diabetes mellitus with unspecified diabetic retinopathy with macular edema |
| E10319 | Type 1 diabetes mellitus with unspecified diabetic retinopathy without macular edema |
| E10321 | Type 1 diabetes mellitus with mild nonproliferative diabetic retinopathy with macular edema |
| E103211 | Type 1 diabetes mellitus with mild nonproliferative diabetic retinopathy with macular edema, right eye |
| E103212 | Type 1 diabetes mellitus with mild nonproliferative diabetic retinopathy with macular edema, left eye |
| E103213 | Type 1 diabetes mellitus with mild nonproliferative diabetic retinopathy with macular edema, bilateral |
| E103219 | Type 1 diabetes mellitus with mild nonproliferative diabetic retinopathy with macular edema, unspecified eye |
| E10329 | Type 1 diabetes mellitus with mild nonproliferative diabetic retinopathy without macular edema |
| E103291 | Type 1 diabetes mellitus with mild nonproliferative diabetic retinopathy without macular edema, right eye |
| E103292 | Type 1 diabetes mellitus with mild nonproliferative diabetic retinopathy without macular edema, left eye |
| E103293 | Type 1 diabetes mellitus with mild nonproliferative diabetic retinopathy without macular edema, bilateral |
| E103299 | Type 1 diabetes mellitus with mild nonproliferative diabetic retinopathy without macular edema, unspecified eye |
| E10331 | Type 1 diabetes mellitus with moderate nonproliferative diabetic retinopathy with macular edema |
| E103311 | Type 1 diabetes mellitus with moderate nonproliferative diabetic retinopathy with macular edema, right eye |
| E103312 | Type 1 diabetes mellitus with moderate nonproliferative diabetic retinopathy with macular edema, left eye |
| E103313 | Type 1 diabetes mellitus with moderate nonproliferative diabetic retinopathy with macular edema, bilateral |
| E103319 | Type 1 diabetes mellitus with moderate nonproliferative diabetic retinopathy with macular edema, unspecified eye |
| E10339 | Type 1 diabetes mellitus with moderate nonproliferative diabetic retinopathy without macular edema |
| E103391 | Type 1 diabetes mellitus with moderate nonproliferative diabetic retinopathy without macular edema, right eye |
| E103392 | Type 1 diabetes mellitus with moderate nonproliferative diabetic retinopathy without macular edema, left eye |
| E103393 | Type 1 diabetes mellitus with moderate nonproliferative diabetic retinopathy without macular edema, bilateral |
| E103399 | Type 1 diabetes mellitus with moderate nonproliferative diabetic retinopathy without macular edema, unspecified eye |
| E10341 | Type 1 diabetes mellitus with severe nonproliferative diabetic retinopathy with macular edema |
| E103411 | Type 1 diabetes mellitus with severe nonproliferative diabetic retinopathy with macular edema, right eye |
| E103412 | Type 1 diabetes mellitus with severe nonproliferative diabetic retinopathy with macular edema, left eye |
| E103413 | Type 1 diabetes mellitus with severe nonproliferative diabetic retinopathy with macular edema, bilateral |
| E103419 | Type 1 diabetes mellitus with severe nonproliferative diabetic retinopathy with macular edema, unspecified eye |
| E10349 | Type 1 diabetes mellitus with severe nonproliferative diabetic retinopathy without macular edema |
| E103491 | Type 1 diabetes mellitus with severe nonproliferative diabetic retinopathy without macular edema, right eye |
| E103492 | Type 1 diabetes mellitus with severe nonproliferative diabetic retinopathy without macular edema, left eye |
| E103493 | Type 1 diabetes mellitus with severe nonproliferative diabetic retinopathy without macular edema, bilateral |
| E103499 | Type 1 diabetes mellitus with severe nonproliferative diabetic retinopathy without macular edema, unspecified eye |
| E10351 | Type 1 diabetes mellitus with proliferative diabetic retinopathy with macular edema |
| E103511 | Type 1 diabetes mellitus with proliferative diabetic retinopathy with macular edema, right eye |
| E103512 | Type 1 diabetes mellitus with proliferative diabetic retinopathy with macular edema, left eye |
| E103513 | Type 1 diabetes mellitus with proliferative diabetic retinopathy with macular edema, bilateral |
| E103519 | Type 1 diabetes mellitus with proliferative diabetic retinopathy with macular edema, unspecified eye |
| E103521 | Type 1 diabetes mellitus with proliferative diabetic retinopathy with traction retinal detachment involving the macula, right eye |
| E103522 | Type 1 diabetes mellitus with proliferative diabetic retinopathy with traction retinal detachment involving the macula, left eye |
| E103523 | Type 1 diabetes mellitus with proliferative diabetic retinopathy with traction retinal detachment involving the macula, bilateral |
| E103529 | Type 1 diabetes mellitus with proliferative diabetic retinopathy with traction retinal detachment involving the macula, unspecified eye |
| E103531 | Type 1 diabetes mellitus with proliferative diabetic retinopathy with traction retinal detachment not involving the macula, right eye |
| E103532 | Type 1 diabetes mellitus with proliferative diabetic retinopathy with traction retinal detachment not involving the macula, left eye |
| E103533 | Type 1 diabetes mellitus with proliferative diabetic retinopathy with traction retinal detachment not involving the macula, bilateral |
| E103539 | Type 1 diabetes mellitus with proliferative diabetic retinopathy with traction retinal detachment not involving the macula, unspecified eye |
| E103541 | Type 1 diabetes mellitus with proliferative diabetic retinopathy with combined traction retinal detachment and rhegmatogenous retinal detachment, right eye |
| E103542 | Type 1 diabetes mellitus with proliferative diabetic retinopathy with combined traction retinal detachment and rhegmatogenous retinal detachment, left eye |
| E103543 | Type 1 diabetes mellitus with proliferative diabetic retinopathy with combined traction retinal detachment and rhegmatogenous retinal detachment, bilateral |
| E103549 | Type 1 diabetes mellitus with proliferative diabetic retinopathy with combined traction retinal detachment and rhegmatogenous retinal detachment, unspecified eye |
| E103551 | Type 1 diabetes mellitus with stable proliferative diabetic retinopathy, right eye |
| E103552 | Type 1 diabetes mellitus with stable proliferative diabetic retinopathy, left eye |
| E103553 | Type 1 diabetes mellitus with stable proliferative diabetic retinopathy, bilateral |
| E103559 | Type 1 diabetes mellitus with stable proliferative diabetic retinopathy, unspecified eye |
| E10359 | Type 1 diabetes mellitus with proliferative diabetic retinopathy without macular edema |
| E103591 | Type 1 diabetes mellitus with proliferative diabetic retinopathy without macular edema, right eye |
| E103592 | Type 1 diabetes mellitus with proliferative diabetic retinopathy without macular edema, left eye |
| E103593 | Type 1 diabetes mellitus with proliferative diabetic retinopathy without macular edema, bilateral |
| E103599 | Type 1 diabetes mellitus with proliferative diabetic retinopathy without macular edema, unspecified eye |
| E1036 | Type 1 diabetes mellitus with diabetic cataract |
| E1037X1 | Type 1 diabetes mellitus with diabetic macular edema, resolved following treatment, right eye |
| E1037X2 | Type 1 diabetes mellitus with diabetic macular edema, resolved following treatment, left eye |
| E1037X3 | Type 1 diabetes mellitus with diabetic macular edema, resolved following treatment, bilateral |
| E1037X9 | Type 1 diabetes mellitus with diabetic macular edema, resolved following treatment, unspecified eye |
| E1039 | Type 1 diabetes mellitus with other diabetic ophthalmic complication |
| E1040 | Type 1 diabetes mellitus with diabetic neuropathy, unspecified |
| E1041 | Type 1 diabetes mellitus with diabetic mononeuropathy |
| E1042 | Type 1 diabetes mellitus with diabetic polyneuropathy |
| E1043 | Type 1 diabetes mellitus with diabetic autonomic (poly)neuropathy |
| E1044 | Type 1 diabetes mellitus with diabetic amyotrophy |
| E1049 | Type 1 diabetes mellitus with other diabetic neurological complication |
| E1051 | Type 1 diabetes mellitus with diabetic peripheral angiopathy without gangrene |
| E1052 | Type 1 diabetes mellitus with diabetic peripheral angiopathy with gangrene |
| E1059 | Type 1 diabetes mellitus with other circulatory complications |
| E10610 | Type 1 diabetes mellitus with diabetic neuropathic arthropathy |
| E10618 | Type 1 diabetes mellitus with other diabetic arthropathy |
| E10620 | Type 1 diabetes mellitus with diabetic dermatitis |
| E10621 | Type 1 diabetes mellitus with foot ulcer |
| E10622 | Type 1 diabetes mellitus with other skin ulcer |
| E10628 | Type 1 diabetes mellitus with other skin complications |
| E10630 | Type 1 diabetes mellitus with periodontal disease |
| E10638 | Type 1 diabetes mellitus with other oral complications |
| E10641 | Type 1 diabetes mellitus with hypoglycemia with coma |
| E10649 | Type 1 diabetes mellitus with hypoglycemia without coma |
| E1065 | Type 1 diabetes mellitus with hyperglycemia |
| E1069 | Type 1 diabetes mellitus with other specified complication |
| E108 | Type 1 diabetes mellitus with unspecified complications |
| E1121 | Type 2 diabetes mellitus with diabetic nephropathy |
| E1122 | Type 2 diabetes mellitus with diabetic chronic kidney disease |
| E1129 | Type 2 diabetes mellitus with other diabetic kidney complication |
| E11311 | Type 2 diabetes mellitus with unspecified diabetic retinopathy with macular edema |
| E11319 | Type 2 diabetes mellitus with unspecified diabetic retinopathy without macular edema |
| E11321 | Type 2 diabetes mellitus with mild nonproliferative diabetic retinopathy with macular edema |
| E113211 | Type 2 diabetes mellitus with mild nonproliferative diabetic retinopathy with macular edema, right eye |
| E113212 | Type 2 diabetes mellitus with mild nonproliferative diabetic retinopathy with macular edema, left eye |
| E113213 | Type 2 diabetes mellitus with mild nonproliferative diabetic retinopathy with macular edema, bilateral |
| E113219 | Type 2 diabetes mellitus with mild nonproliferative diabetic retinopathy with macular edema, unspecified eye |
| E11329 | Type 2 diabetes mellitus with mild nonproliferative diabetic retinopathy without macular edema |
| E113291 | Type 2 diabetes mellitus with mild nonproliferative diabetic retinopathy without macular edema, right eye |
| E113292 | Type 2 diabetes mellitus with mild nonproliferative diabetic retinopathy without macular edema, left eye |
| E113293 | Type 2 diabetes mellitus with mild nonproliferative diabetic retinopathy without macular edema, bilateral |
| E113299 | Type 2 diabetes mellitus with mild nonproliferative diabetic retinopathy without macular edema, unspecified eye |
| E11331 | Type 2 diabetes mellitus with moderate nonproliferative diabetic retinopathy with macular edema |
| E113311 | Type 2 diabetes mellitus with moderate nonproliferative diabetic retinopathy with macular edema, right eye |
| E113312 | Type 2 diabetes mellitus with moderate nonproliferative diabetic retinopathy with macular edema, left eye |
| E113313 | Type 2 diabetes mellitus with moderate nonproliferative diabetic retinopathy with macular edema, bilateral |
| E113319 | Type 2 diabetes mellitus with moderate nonproliferative diabetic retinopathy with macular edema, unspecified eye |
| E11339 | Type 2 diabetes mellitus with moderate nonproliferative diabetic retinopathy without macular edema |
| E113391 | Type 2 diabetes mellitus with moderate nonproliferative diabetic retinopathy without macular edema, right eye |
| E113392 | Type 2 diabetes mellitus with moderate nonproliferative diabetic retinopathy without macular edema, left eye |
| E113393 | Type 2 diabetes mellitus with moderate nonproliferative diabetic retinopathy without macular edema, bilateral |
| E113399 | Type 2 diabetes mellitus with moderate nonproliferative diabetic retinopathy without macular edema, unspecified eye |
| E11341 | Type 2 diabetes mellitus with severe nonproliferative diabetic retinopathy with macular edema |
| E113411 | Type 2 diabetes mellitus with severe nonproliferative diabetic retinopathy with macular edema, right eye |
| E113412 | Type 2 diabetes mellitus with severe nonproliferative diabetic retinopathy with macular edema, left eye |
| E113413 | Type 2 diabetes mellitus with severe nonproliferative diabetic retinopathy with macular edema, bilateral |
| E113419 | Type 2 diabetes mellitus with severe nonproliferative diabetic retinopathy with macular edema, unspecified eye |
| E11349 | Type 2 diabetes mellitus with severe nonproliferative diabetic retinopathy without macular edema |
| E113491 | Type 2 diabetes mellitus with severe nonproliferative diabetic retinopathy without macular edema, right eye |
| E113492 | Type 2 diabetes mellitus with severe nonproliferative diabetic retinopathy without macular edema, left eye |
| E113493 | Type 2 diabetes mellitus with severe nonproliferative diabetic retinopathy without macular edema, bilateral |
| E113499 | Type 2 diabetes mellitus with severe nonproliferative diabetic retinopathy without macular edema, unspecified eye |
| E11351 | Type 2 diabetes mellitus with proliferative diabetic retinopathy with macular edema |
| E113511 | Type 2 diabetes mellitus with proliferative diabetic retinopathy with macular edema, right eye |
| E113512 | Type 2 diabetes mellitus with proliferative diabetic retinopathy with macular edema, left eye |
| E113513 | Type 2 diabetes mellitus with proliferative diabetic retinopathy with macular edema, bilateral |
| E113519 | Type 2 diabetes mellitus with proliferative diabetic retinopathy with macular edema, unspecified eye |
| E113521 | Type 2 diabetes mellitus with proliferative diabetic retinopathy with traction retinal detachment involving the macula, right eye |
| E113522 | Type 2 diabetes mellitus with proliferative diabetic retinopathy with traction retinal detachment involving the macula, left eye |
| E113523 | Type 2 diabetes mellitus with proliferative diabetic retinopathy with traction retinal detachment involving the macula, bilateral |
| E113529 | Type 2 diabetes mellitus with proliferative diabetic retinopathy with traction retinal detachment involving the macula, unspecified eye |
| E113531 | Type 2 diabetes mellitus with proliferative diabetic retinopathy with traction retinal detachment not involving the macula, right eye |
| E113532 | Type 2 diabetes mellitus with proliferative diabetic retinopathy with traction retinal detachment not involving the macula, left eye |
| E113533 | Type 2 diabetes mellitus with proliferative diabetic retinopathy with traction retinal detachment not involving the macula, bilateral |
| E113539 | Type 2 diabetes mellitus with proliferative diabetic retinopathy with traction retinal detachment not involving the macula, unspecified eye |
| E113541 | Type 2 diabetes mellitus with proliferative diabetic retinopathy with combined traction retinal detachment and rhegmatogenous retinal detachment, right eye |
| E113542 | Type 2 diabetes mellitus with proliferative diabetic retinopathy with combined traction retinal detachment and rhegmatogenous retinal detachment, left eye |
| E113543 | Type 2 diabetes mellitus with proliferative diabetic retinopathy with combined traction retinal detachment and rhegmatogenous retinal detachment, bilateral |
| E113549 | Type 2 diabetes mellitus with proliferative diabetic retinopathy with combined traction retinal detachment and rhegmatogenous retinal detachment, unspecified eye |
| E113551 | Type 2 diabetes mellitus with stable proliferative diabetic retinopathy, right eye |
| E113552 | Type 2 diabetes mellitus with stable proliferative diabetic retinopathy, left eye |
| E113553 | Type 2 diabetes mellitus with stable proliferative diabetic retinopathy, bilateral |
| E113559 | Type 2 diabetes mellitus with stable proliferative diabetic retinopathy, unspecified eye |
| E11359 | Type 2 diabetes mellitus with proliferative diabetic retinopathy without macular edema |
| E113591 | Type 2 diabetes mellitus with proliferative diabetic retinopathy without macular edema, right eye |
| E113592 | Type 2 diabetes mellitus with proliferative diabetic retinopathy without macular edema, left eye |
| E113593 | Type 2 diabetes mellitus with proliferative diabetic retinopathy without macular edema, bilateral |
| E113599 | Type 2 diabetes mellitus with proliferative diabetic retinopathy without macular edema, unspecified eye |
| E1136 | Type 2 diabetes mellitus with diabetic cataract |
| E1137X1 | Type 2 diabetes mellitus with diabetic macular edema, resolved following treatment, right eye |
| E1137X2 | Type 2 diabetes mellitus with diabetic macular edema, resolved following treatment, left eye |
| E1137X3 | Type 2 diabetes mellitus with diabetic macular edema, resolved following treatment, bilateral |
| E1137X9 | Type 2 diabetes mellitus with diabetic macular edema, resolved following treatment, unspecified eye |
| E1139 | Type 2 diabetes mellitus with other diabetic ophthalmic complication |
| E1140 | Type 2 diabetes mellitus with diabetic neuropathy, unspecified |
| E1141 | Type 2 diabetes mellitus with diabetic mononeuropathy |
| E1142 | Type 2 diabetes mellitus with diabetic polyneuropathy |
| E1143 | Type 2 diabetes mellitus with diabetic autonomic (poly)neuropathy |
| E1144 | Type 2 diabetes mellitus with diabetic amyotrophy |
| E1149 | Type 2 diabetes mellitus with other diabetic neurological complication |
| E1151 | Type 2 diabetes mellitus with diabetic peripheral angiopathy without gangrene |
| E1152 | Type 2 diabetes mellitus with diabetic peripheral angiopathy with gangrene |
| E1159 | Type 2 diabetes mellitus with other circulatory complications |
| E11610 | Type 2 diabetes mellitus with diabetic neuropathic arthropathy |
| E11618 | Type 2 diabetes mellitus with other diabetic arthropathy |
| E11620 | Type 2 diabetes mellitus with diabetic dermatitis |
| E11621 | Type 2 diabetes mellitus with foot ulcer |
| E11622 | Type 2 diabetes mellitus with other skin ulcer |
| E11628 | Type 2 diabetes mellitus with other skin complications |
| E11630 | Type 2 diabetes mellitus with periodontal disease |
| E11638 | Type 2 diabetes mellitus with other oral complications |
| E11641 | Type 2 diabetes mellitus with hypoglycemia with coma |
| E11649 | Type 2 diabetes mellitus with hypoglycemia without coma |
| E1165 | Type 2 diabetes mellitus with hyperglycemia |
| E1169 | Type 2 diabetes mellitus with other specified complication |
| E118 | Type 2 diabetes mellitus with unspecified complications |
| E1321 | Other specified diabetes mellitus with diabetic nephropathy |
| E1322 | Other specified diabetes mellitus with diabetic chronic kidney disease |
| E1329 | Other specified diabetes mellitus with other diabetic kidney complication |
| E13311 | Other specified diabetes mellitus with unspecified diabetic retinopathy with macular edema |
| E13319 | Other specified diabetes mellitus with unspecified diabetic retinopathy without macular edema |
| E13321 | Other specified diabetes mellitus with mild nonproliferative diabetic retinopathy with macular edema |
| E133211 | Other specified diabetes mellitus with mild nonproliferative diabetic retinopathy with macular edema, right eye |
| E133212 | Other specified diabetes mellitus with mild nonproliferative diabetic retinopathy with macular edema, left eye |
| E133213 | Other specified diabetes mellitus with mild nonproliferative diabetic retinopathy with macular edema, bilateral |
| E133219 | Other specified diabetes mellitus with mild nonproliferative diabetic retinopathy with macular edema, unspecified eye |
| E13329 | Other specified diabetes mellitus with mild nonproliferative diabetic retinopathy without macular edema |
| E133291 | Other specified diabetes mellitus with mild nonproliferative diabetic retinopathy without macular edema, right eye |
| E133292 | Other specified diabetes mellitus with mild nonproliferative diabetic retinopathy without macular edema, left eye |
| E133293 | Other specified diabetes mellitus with mild nonproliferative diabetic retinopathy without macular edema, bilateral |
| E133299 | Other specified diabetes mellitus with mild nonproliferative diabetic retinopathy without macular edema, unspecified eye |
| E13331 | Other specified diabetes mellitus with moderate nonproliferative diabetic retinopathy with macular edema |
| E133311 | Other specified diabetes mellitus with moderate nonproliferative diabetic retinopathy with macular edema, right eye |
| E133312 | Other specified diabetes mellitus with moderate nonproliferative diabetic retinopathy with macular edema, left eye |
| E133313 | Other specified diabetes mellitus with moderate nonproliferative diabetic retinopathy with macular edema, bilateral |
| E133319 | Other specified diabetes mellitus with moderate nonproliferative diabetic retinopathy with macular edema, unspecified eye |
| E13339 | Other specified diabetes mellitus with moderate nonproliferative diabetic retinopathy without macular edema |
| E133391 | Other specified diabetes mellitus with moderate nonproliferative diabetic retinopathy without macular edema, right eye |
| E133392 | Other specified diabetes mellitus with moderate nonproliferative diabetic retinopathy without macular edema, left eye |
| E133393 | Other specified diabetes mellitus with moderate nonproliferative diabetic retinopathy without macular edema, bilateral |
| E133399 | Other specified diabetes mellitus with moderate nonproliferative diabetic retinopathy without macular edema, unspecified eye |
| E13341 | Other specified diabetes mellitus with severe nonproliferative diabetic retinopathy with macular edema |
| E133411 | Other specified diabetes mellitus with severe nonproliferative diabetic retinopathy with macular edema, right eye |
| E133412 | Other specified diabetes mellitus with severe nonproliferative diabetic retinopathy with macular edema, left eye |
| E133413 | Other specified diabetes mellitus with severe nonproliferative diabetic retinopathy with macular edema, bilateral |
| E133419 | Other specified diabetes mellitus with severe nonproliferative diabetic retinopathy with macular edema, unspecified eye |
| E13349 | Other specified diabetes mellitus with severe nonproliferative diabetic retinopathy without macular edema |
| E133491 | Other specified diabetes mellitus with severe nonproliferative diabetic retinopathy without macular edema, right eye |
| E133492 | Other specified diabetes mellitus with severe nonproliferative diabetic retinopathy without macular edema, left eye |
| E133493 | Other specified diabetes mellitus with severe nonproliferative diabetic retinopathy without macular edema, bilateral |
| E133499 | Other specified diabetes mellitus with severe nonproliferative diabetic retinopathy without macular edema, unspecified eye |
| E13351 | Other specified diabetes mellitus with proliferative diabetic retinopathy with macular edema |
| E133511 | Other specified diabetes mellitus with proliferative diabetic retinopathy with macular edema, right eye |
| E133512 | Other specified diabetes mellitus with proliferative diabetic retinopathy with macular edema, left eye |
| E133513 | Other specified diabetes mellitus with proliferative diabetic retinopathy with macular edema, bilateral |
| E133519 | Other specified diabetes mellitus with proliferative diabetic retinopathy with macular edema, unspecified eye |
| E133521 | Other specified diabetes mellitus with proliferative diabetic retinopathy with traction retinal detachment involving the macula, right eye |
| E133522 | Other specified diabetes mellitus with proliferative diabetic retinopathy with traction retinal detachment involving the macula, left eye |
| E133523 | Other specified diabetes mellitus with proliferative diabetic retinopathy with traction retinal detachment involving the macula, bilateral |
| E133529 | Other specified diabetes mellitus with proliferative diabetic retinopathy with traction retinal detachment involving the macula, unspecified eye |
| E133531 | Other specified diabetes mellitus with proliferative diabetic retinopathy with traction retinal detachment not involving the macula, right eye |
| E133532 | Other specified diabetes mellitus with proliferative diabetic retinopathy with traction retinal detachment not involving the macula, left eye |
| E133533 | Other specified diabetes mellitus with proliferative diabetic retinopathy with traction retinal detachment not involving the macula, bilateral |
| E133539 | Other specified diabetes mellitus with proliferative diabetic retinopathy with traction retinal detachment not involving the macula, unspecified eye |
| E133541 | Other specified diabetes mellitus with proliferative diabetic retinopathy with combined traction retinal detachment and rhegmatogenous retinal detachment, right eye |
| E133542 | Other specified diabetes mellitus with proliferative diabetic retinopathy with combined traction retinal detachment and rhegmatogenous retinal detachment, left eye |
| E133543 | Other specified diabetes mellitus with proliferative diabetic retinopathy with combined traction retinal detachment and rhegmatogenous retinal detachment, bilateral |
| E133549 | Other specified diabetes mellitus with proliferative diabetic retinopathy with combined traction retinal detachment and rhegmatogenous retinal detachment, unspecified eye |
| E133551 | Other specified diabetes mellitus with stable proliferative diabetic retinopathy, right eye |
| E133552 | Other specified diabetes mellitus with stable proliferative diabetic retinopathy, left eye |
| E133553 | Other specified diabetes mellitus with stable proliferative diabetic retinopathy, bilateral |
| E133559 | Other specified diabetes mellitus with stable proliferative diabetic retinopathy, unspecified eye |
| E13359 | Other specified diabetes mellitus with proliferative diabetic retinopathy without macular edema |
| E133591 | Other specified diabetes mellitus with proliferative diabetic retinopathy without macular edema, right eye |
| E133592 | Other specified diabetes mellitus with proliferative diabetic retinopathy without macular edema, left eye |
| E133593 | Other specified diabetes mellitus with proliferative diabetic retinopathy without macular edema, bilateral |
| E133599 | Other specified diabetes mellitus with proliferative diabetic retinopathy without macular edema, unspecified eye |
| E1336 | Other specified diabetes mellitus with diabetic cataract |
| E1337X1 | Other specified diabetes mellitus with diabetic macular edema, resolved following treatment, right eye |
| E1337X2 | Other specified diabetes mellitus with diabetic macular edema, resolved following treatment, left eye |
| E1337X3 | Other specified diabetes mellitus with diabetic macular edema, resolved following treatment, bilateral |
| E1337X9 | Other specified diabetes mellitus with diabetic macular edema, resolved following treatment, unspecified eye |
| E1339 | Other specified diabetes mellitus with other diabetic ophthalmic complication |
| E1340 | Other specified diabetes mellitus with diabetic neuropathy, unspecified |
| E1341 | Other specified diabetes mellitus with diabetic mononeuropathy |
| E1342 | Other specified diabetes mellitus with diabetic polyneuropathy |
| E1343 | Other specified diabetes mellitus with diabetic autonomic (poly)neuropathy |
| E1344 | Other specified diabetes mellitus with diabetic amyotrophy |
| E1349 | Other specified diabetes mellitus with other diabetic neurological complication |
| E1351 | Other specified diabetes mellitus with diabetic peripheral angiopathy without gangrene |
| E1352 | Other specified diabetes mellitus with diabetic peripheral angiopathy with gangrene |
| E1359 | Other specified diabetes mellitus with other circulatory complications |
| E13610 | Other specified diabetes mellitus with diabetic neuropathic arthropathy |
| E13618 | Other specified diabetes mellitus with other diabetic arthropathy |
| E13620 | Other specified diabetes mellitus with diabetic dermatitis |
| E13621 | Other specified diabetes mellitus with foot ulcer |
| E13622 | Other specified diabetes mellitus with other skin ulcer |
| E13628 | Other specified diabetes mellitus with other skin complications |
| E13630 | Other specified diabetes mellitus with periodontal disease |
| E13638 | Other specified diabetes mellitus with other oral complications |
| E13641 | Other specified diabetes mellitus with hypoglycemia with coma |
| E13649 | Other specified diabetes mellitus with hypoglycemia without coma |
| E1365 | Other specified diabetes mellitus with hyperglycemia |
| E1369 | Other specified diabetes mellitus with other specified complication |
| E138 | Other specified diabetes mellitus with unspecified complications |
